# Genome editing by introduction of Cas9/sgRNA into plant cells using temperature-controlled atmospheric pressure plasma

**DOI:** 10.1371/journal.pone.0281767

**Published:** 2023-02-16

**Authors:** Yuki Yanagawa, Yuma Suenaga, Yusuke Iijima, Masaki Endo, Naoko Sanada, Etsuko Katoh, Seiichi Toki, Akitoshi Okino, Ichiro Mitsuhara

**Affiliations:** 1 Graduate School of Horticulture, Chiba University, Matsudo, Chiba, Japan; 2 Institute of Agrobiological Sciences, NARO, Tsukuba, Ibaraki, Japan; 3 RIKEN Center for Sustainable Resource Science, Yokohama, Kanagawa, Japan; 4 Laboratory for Future Interdisciplinary Research of Science and Technology, Institute of Innovative Research, Tokyo Institute of Technology, Yokohama, Kanagawa, Japan; 5 Graduate School of Nanobioscience, Yokohama City University, Yokohama, Kanagawa, Japan; 6 Kihara Institute for Biological Research, Yokohama City University, Yokohama, Kanagawa, Japan; 7 Advanced Analysis Center, NARO, Tsukuba, Ibaraki, Japan; 8 Faculty of Food and Nutritional Science, Toyo University, Ora-gun, Gunma, Japan; 9 Faculty of Agriculture, Ryukoku University, Otsu, Shiga, Japan; University of Tsukuba, JAPAN

## Abstract

Previously, we developed a technique to introduce a superfolder green fluorescent protein (sGFP) fusion protein directly into plant cells using atmospheric-pressure plasma. In this study, we attempted genome editing using CRISPR/Cas9 (clustered regularly interspaced short palindromic repeats/CRISPR associated protein 9) system using this protein introduction technique. As an experimental system to evaluate genome editing, we utilized transgenic reporter plants carrying the reporter genes *L-(I-SceI)-UC* and *sGFP-waxy-HPT*. The L-(I-SceI)-UC system allowed the detection of successful genome editing by measuring the chemiluminescent signal observed upon re-functionalization of the luciferase (*LUC*) gene following genome editing. Similarly, the sGFP-waxy-HPT system conferred hygromycin resistance caused by hygromycin phosphotransferase (HPT) during genome editing. CRISPR/Cas9 ribonucleoproteins targeting these reporter genes were directly introduced into rice calli or tobacco leaf pieces after treatment with N_2_ and/or CO_2_ plasma. Cultivation of the treated rice calli on a suitable medium plate produced the luminescence signal, which was not observed in the negative control. Four types of genome-edited sequences were obtained upon sequencing the reporter genes of genome-edited candidate calli. *sGFP-waxy-HPT*-carrying tobacco cells exhibited hygromycin resistance during genome editing. After repeated cultivation of the treated tobacco leaf pieces on a regeneration medium plate, the calli were observed with leaf pieces. A green callus that was hygromycin-resistant was harvested, and a genome-edited sequence in the tobacco reporter gene was confirmed. As direct introduction of the Cas9/sgRNA (single guide RNA) complex using plasma enables genome editing in plants without any DNA introduction, this method is expected to be optimized for many plant species and may be widely applied for plant breeding in the future.

## Introduction

Genome editing is employed as one of strategies for plant breeding. It is used to inactivate or modify target genes by introducing a mutation or insertion in the sequences of certain genes in the genome using genome editing enzymes that are composed of proteins such as zinc-finger nucleases and transcription activator-like effector nucleases (TALENs) or protein-RNA complexes, such as CRISPR/Cas9 system [1–5]. Different from genetically modified organisms (GMO), which comprise exogenous genes inserted in the genome, genome-edited organisms show mutations within endogenous genes. In mammalian cells, genome editing enzymes can be easily introduced by several methods such as transfection, which involves introduction of macromolecules into eukaryotes using various delivery vehicles [6, 7]. However, direct introduction of genome editing enzymes is difficult in plants because intact plant tissues have several barriers, such as the wax layer, cuticular layer, and cell wall. Therefore, in most plants, genes encoding specific genome editing enzymes are introduced via genetic transformation, and the introduced genes are removed after genome editing. Recently, several methods have been reported for introduction of proteins into plant cells, such as the use of a cell penetrating peptide (CPP) [8, 9], in planta particle bombardment [10], and protein transportation by a bacterium carrying a type III secretion system [11]. Although they work for genome editing enzymes, they have limitations with respect to the tissues where they can be used, such as shoot meristems, host plants against bacterial infection, and/or the need for pretreatment for protein introduction [10–12]. Previously, we developed a method for introduction of proteins into plant cells of intact leaves and roots without any pretreatment using temperature-controlled atmospheric-pressure plasma [13]. Therefore, the plasma method can possibly be used for the introduction of genome editing enzymes in various plant species and plant tissues. In mammalian cells, genome editing has been performed by introducing Cas9/single guide (sg) RNA into the cells using atmospheric-pressure plasma generated by argon (Ar) [14]. This result was obtained using mammalian cells. Our previous results revealed that plasma treatment can introduce macromolecules into intact plant tissues [13]. Therefore, we attempted genome editing by introducing Cas9/sgRNA in plants using atmospheric-pressure plasma.

In our experiments, we used rice calli and tobacco leaf pieces carrying reporter genes to evaluate genome editing. By Cas9/sgRNA introduction using plasma treatment, genome-edited sequences were obtained from both rice and tobacco cells introduced with Cas9/sgRNA following treatment with temperature-controlled plasma. Here, we suggest that our method performed using temperature-controlled atmospheric-pressure plasma is useful for genome editing following introduction of Cas9/sgRNA into various plant species and/or tissues.

## Materials and methods

### Materials, growth conditions of plants, and primers and oligos

Rice (*Oryza sativa* Nipponbare) calli were grown in a growth chamber at 28°C under a 16 h/8 h light/dark cycle. N6D medium plate [1× Chu (N6) medium salt mixture (Fujifilm, Osaka, Japan), 2 mg/L glycine, 0.5 mg/L nicotine, 0.5 mg/L pyridoxine hydrochloride, 1 mg/L thiamine hydrochloride, 2.9 g/L L-proline, 0.3 g/L casamino acid, 30 g/L sucrose, 0.1 mg/L 2-naphthoic acid, and 4 g/L gellite, pH 5.8] was used to cultivate them. Tobacco plants (*Nicotiana tabacum* cv. Samsun NN) used in this study were planted in a pot with vermiculite and grown in a growth room at 25°C under a 16 h/8 h light/dark cycle.

Information on all primers and oligos used in this study is presented in S1 Table.

### Vector construction and preparation of reporter plants

The reporter construct of the *LUC* gene interrupted by a recognition sequence of a homing endonuclease was introduced into rice calli as described previously [15]. To develop reporter tobacco plants for genome editing, reporter constructs composed of genes encoding sGFP and hygromycin phosphotransferase (HPT) were constructed. In the construct, the open reading frames (ORFs) for sGFP and HPT were connected by a short fragment of the rice *waxy* gene [16]; however, these ORFs were not arranged for translational fusion of sGFP and HPT. Hence, the developed construct could not function as a hygromycin-resistant gene. A DNA fragment comprising a short sequence of the *waxy* gene was produced by annealing two single-stranded DNAs, XbaI-waxy-EcoRI-F, and EcoRI-waxy-XbaI-R sequences. It was then inserted into the pEl2Ω-MCS vector digested with *Xba*I and *Eco*RI, leading to the development of pEL2Ω-waxy vector. The sequence of the *HPT* gene was amplified using the cHPT-F and SpeI-HPT-R primers. The resulting fragment was digested with *Spe*I and inserted into the pEL2Ω-waxy vector digested with *Eco*RI and *Spe*I. The resulting pEL2Ω-waxy-HPT vector was digested with *Xba*I and *Sac*I and then inserted into pBI121 digested with *Xba*I and *Sac*I to produce pBI121-waxy-HPT. *sGFP* DNA fragment was amplified using the primers XbaI-sGFP-F and XbaI-sGFP-R and then digested with *Xba*I. The *sGFP* fragment was inserted into pBI121-waxy-HPT and digested with *Xba*I to produce pBI121-sGFP-waxy-HPT. The resulting construct was introduced into tobacco plants by *Agrobacterium-*mediated transformation as described [17]. Second-generation sGFP-waxy-HPT plants were used in this study.

### Preparation of Cas9/sgRNA

Cas9 protein was prepared as described previously [18]. To prepare sgRNA for L-(I-SceI)-UC and sGFP-waxy-HPT reporter plants, the DNA fragment was amplified using the primers Guide-it scaffold Template and I-SceI-LUC Guide it or GFP-wx-Guide it, respectively. Each sgRNA was prepared using the ScriptMAX^Ⓡ^ Thermo T7 Transcription kit (TOYOBO, Osaka, Japan) according to the manufacturer’s instructions. To prepare the Cas9/sgRNA complex, Cas9 and sgRNA were mixed in a 5:1 weight ratio and incubated in 1× NEB3 buffer at 4°C for 30 min.

### Plasma treatment, introduction of Cas9/sgRNA into cells, and generation of genome-edited cells

**Fig 1:**
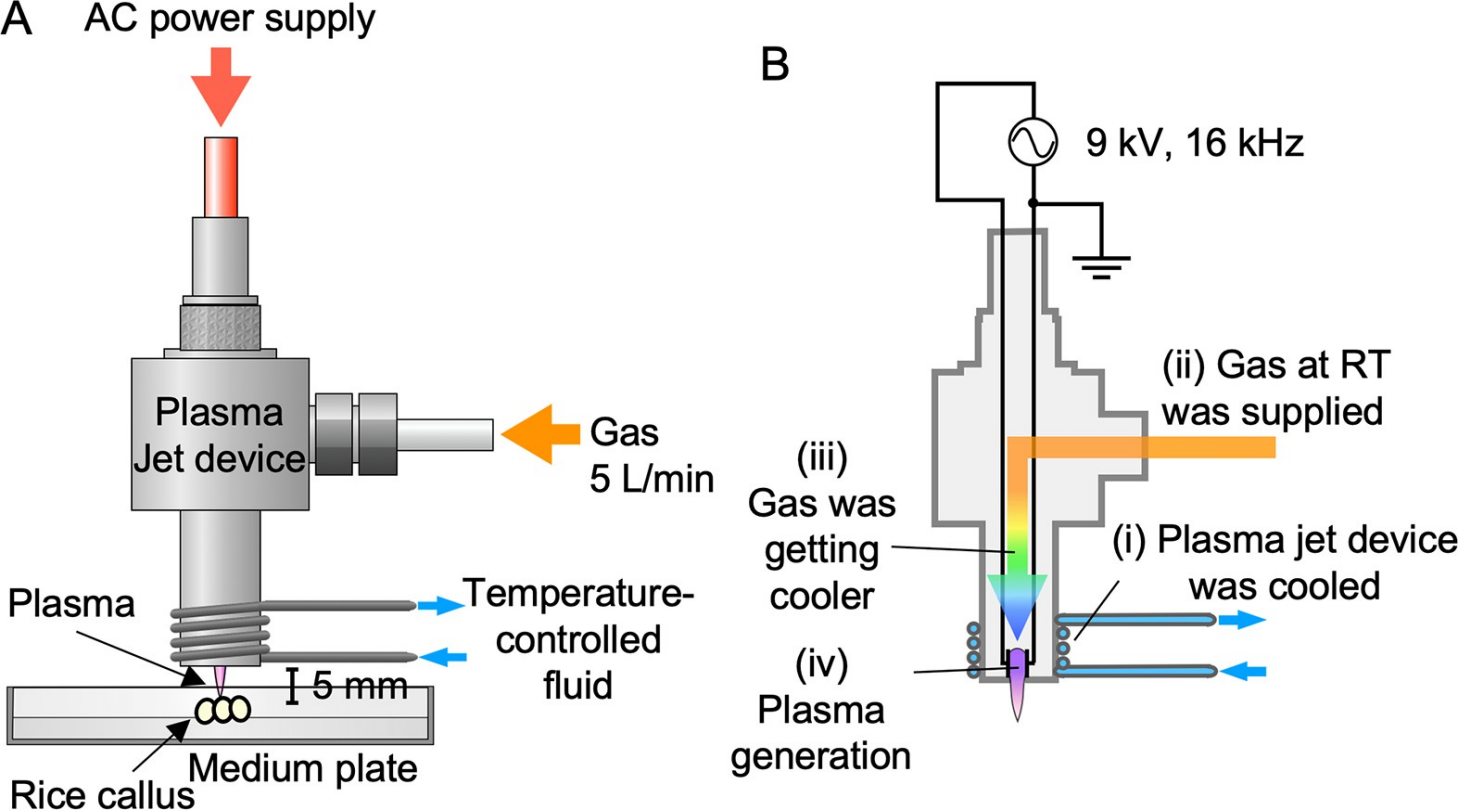
Plasma treatment by a multi-gas temperature controllable plasma jet. (A) Schematic image of plasma irradiation of plant tissues. Rice calli placed on a medium plate were irradiated with plasma at a distance of 5 mm. (B) Scheme for temperature-controlled plasma generation. The device producing the multi-gas temperature-controllable plasma jet was cooled by temperature-controlled fluid (i). N_2_ or CO_2_ at room temperature (RT) was supplied to a metal tube outside the device (ii). The gas was cooled via heat exchange (iii). The cooled gas flowed between the internal high-voltage electrodes upon application of AC voltage, generating room-temperature plasma (20–30°C) by discharge (iv).

Rice calli and tobacco leaf pieces were treated using a multi-gas temperature-controllable plasma jet, as shown in Fig 1. The plasma jet device was grounded, and the internal high-voltage electrode was connected to an AC power supply (Plasma Concept Tokyo Inc.) of 16 kHz and 9 kV at approximately 10 W. Temperature-controlled atmospheric-pressure plasma was generated from nitrogen (N_2_) or carbon dioxide (CO_2_) and cooled by a gas-cooling system using a temperature-controlled fluid. The plasma was ejected from a 1 mm aperture of the plasma jet device at a flow rate of 5 L/min [19–21].

For genome editing of L-(I-SceI)-UC rice cells, the calli were treated with N_2_ or CO_2_ plasma for 5 sec at a distance of 5 mm from the outlet of the plasma jet device. After plasma treatment, the calli were soaked in a 24-well culture plate with 500 μL/well of buffer containing 1/4× phosphate-buffered saline (PBS) and 10 mM MgCl_2_ with Cas9 (50 μg/mL)/sgRNA (10 μg/mL) or 50 μg/mL bovine serum albumin (BSA), followed by incubation at room temperature overnight. The calli were transferred onto an N6D medium plate and maintained at 28°C. After cultivation of the calli for appropriate period, 10 mM phosphate buffer (pH 7.0) containing 1 mM luciferin was sprayed onto the calli, and chemiluminescence of LUC was monitored using an LAS-3000 imager analyzer (Fujifilm).

For genome editing of the sGFP-waxy-HPT-carrying tobacco cells, tobacco leaves were sterilized with 1% hypochlorous acid for 2 min and cut into squares of sides approximately 2 cm. The leaf pieces were kept on 1/2× Murashige and Skoog medium (MS) medium plate [1/2× MS, 8.5 g/L agar, pH 5.8] for 1 day. N_2_ plasma was applied to the leaf pieces at a distance of 5 mm from the outlet of the plasma jet device. The plasma-treated leaf piece was placed in a 12-well culture plate with 400 μL/well of buffer containing 1/4× PBS and 10 mM MgCl_2_ with Cas9 (50 μg/mL)/sgRNA (10 μg/mL) or 50 μg/mL BSA and then incubated at room temperature overnight. The leaf piece was transferred on a regeneration medium plate [1×MS, 1× MS vitamin (0.1 μg/ml thiamine hydrochloride, 0.5 μg/mL pyridoxine hydrochloride, 0.5 μg/mL nicotinamide, 2 μg/mL glycine, 100 μg/mL myo-inositol), 0.1 μg/mL α-naphthaleneacetic acid, 1 μg/mL 6-benzylaminopurine, 30 g/L sucrose, 8.5 g/L agar, pH 5.8] and kept at 28°C for 2 days. To obtain genome-edited cells, a leaf piece was cut into 4 pieces, transferred to a regeneration medium plate containing 50 μg/mL of hygromycin, and stored at 28°C. The leaf pieces were transferred to a new regeneration medium plate containing 50 μg/mL of hygromycin every week to select genome-edited cells.

### Verification of genome editing

Genomic DNA was prepared from genome-edited rice calli and tobacco cells using DNA suisui-P kit according to the manufacturer’s instructions (Rizo Inc., Ibaraki, Japan). For rice, a fragment including the Cas9 target site was amplified from the rice genomic DNA by PrimeSTAR Max DNA polymerase (Takara Bio, Shiga, Japan) using primers P35s 90–5 and ELuc-217R. PCR products were digested using I-*Sce*I. For tobacco, a fragment including the Cas9 target site was amplified from the tobacco genomic DNA using primers sGFP-441F and HPT3. The PCR product was digested with Cas9/sgRNA. The digested samples containing I-*Sce*I or Cas9/sgRNA-resistant components were cloned using the Zero blunt^Ⓡ^ TOPO^Ⓡ^ PCR cloning kit (Thermo fisher scientific, MA, USA) and transformed into the *Escherichia coli* strain DH5α. Plasmid DNA was prepared from the bacterial colonies, and the predicted genome-edited regions were sequenced.

Next generation plants were regenerated from the genome-edited tobacco callus and their seeds were harvested. To examine hygromycin resistance, the seeds were planted on 1/2× MS medium plates [1/2× MS, 8.5 g/L agar, pH 5.8] containing 50 μg/mL of hygromycin and kept at 28°C for 2 weeks.

## Results

### Genome editing in rice calli carrying a reporter gene

**Fig 2:**
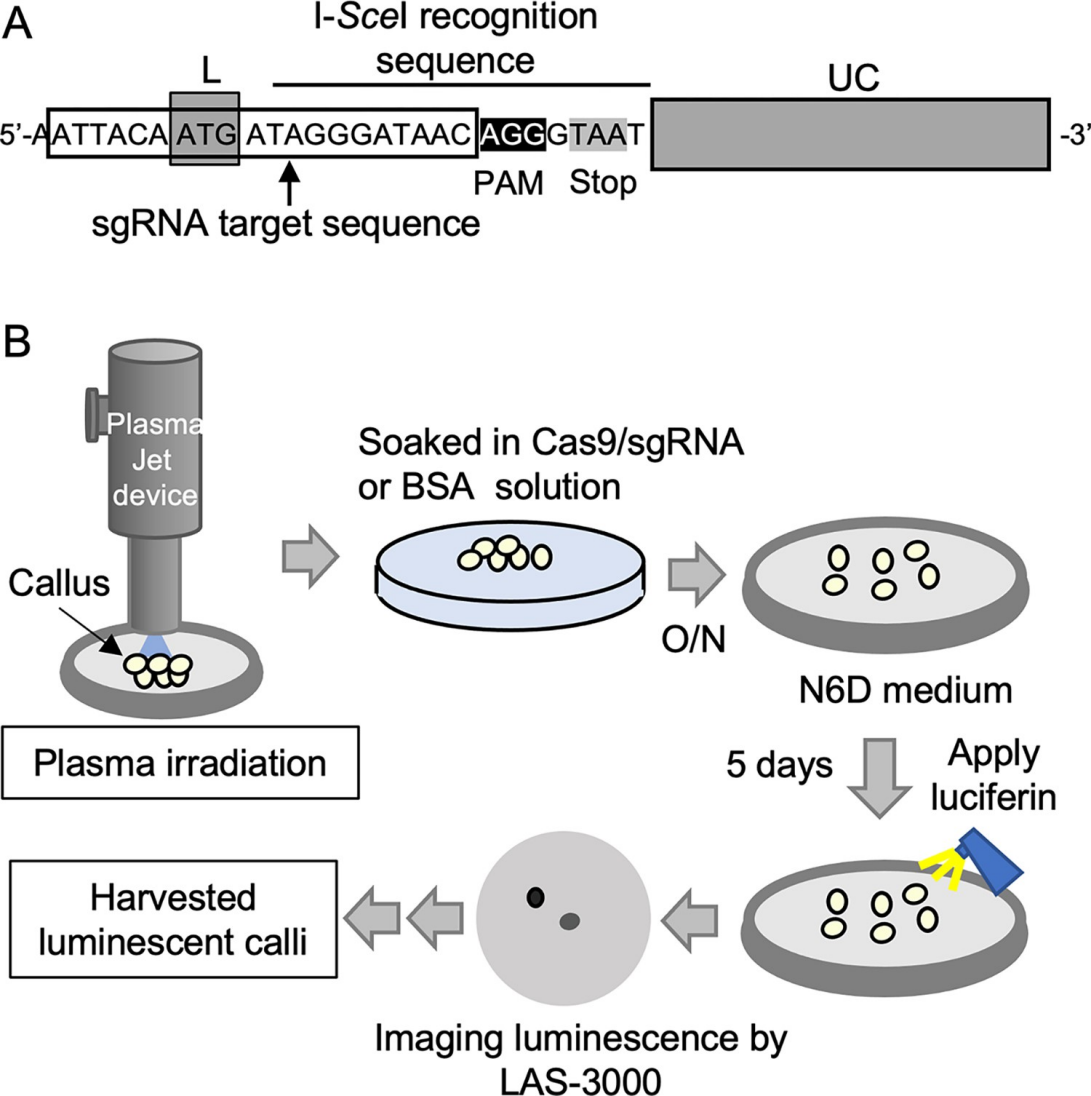
Rice reporter plant and procedure of the plasma treatment. (A) The structure of the *L-(I-SceI)-UC* reporter gene. The *LUC* gene was interrupted by the I-*Sce*I recognition sequence. sgRNA target sequence was designated as that comprising 20 nucleotides located immediately upstream of PAM sequence (AGG). Cas9/sgRNA digested at a site located approximately 3 to 4 base pairs upstream of PAM sequence. The *LUC* construct is inactive because the first ATG codon and *LUC* ORF was not located in-frame, and the translation was immediately terminated at stop codon (TAA) located just downstream of I-*Sce*I recognition sequence. *LUC* sequence can be changed in-frame when a frameshift occurs upon misrepair after digestion by Cas9/sgRNA, followed by demonstration of LUC chemiluminescence. (B) Scheme of generating genome-edited rice calli by introducing Cas9/sgRNA into the cells using plasma. Rice calli harboring the reporter gene were irradiated by N_2_ or CO_2_ plasma. The treated calli were soaked into Cas9/sgRNA or BSA solution and incubated overnight (O/N). After the calli were maintained for 5 days on the N6D medium plate, luciferin was sprayed to monitor the luminescent calli. The luminescent calli were cultured for 1 month and harvested.

Previously, we developed a technique for protein introduction using irradiation of plasma generated by N_2_ or CO_2_ to plant tissues, but not N_2_ nor CO_2_ gas [13]. Using this technique, we attempted to edit the genome of rice by introducing a genome editing enzyme complex Cas9/sgRNA. To prove this concept, rice calli containing the reporter gene *L-(I-SceI)-UC* were developed to detect genome editing (Fig 2A). LUC is an enzyme that catalyzes the oxidation of luciferin which is accompanied by the production of light. The introduced *L-(I-SceI)-UC* gene was nonfunctional because of the insertion of a fragment containing the I-*Sce*I recognition sequence. Translation was initiated at the first ATG codon of the reporter construct and was immediately terminated at a stop codon in the inserted fragment, which was located in-frame of the first ATG codon. Thus, the calli from the plant introduced with the reporter construct did not exhibit chemiluminescence. Cells have repair mechanisms for the repair of double strand breaks; however, misrepair often occurs in many organisms. Cas9/sgRNA digests at a site located approximately 3 to 4 base pairs upstream of the PAM (Proto-spacer Adjacent Motif) sequence. Repair errors occurred at the inserted sequence after Cas9/sgRNA digestion, resulting in an in-frame shift of *LUC* gene in addition to the out-of-frame. Since the translation of in-frame *LUC* gene led to normal LUC protein with chemiluminescent ability, some genome-edited rice cells could be observed upon application of luciferin owing to the luminescence signal.

**Fig 3:**
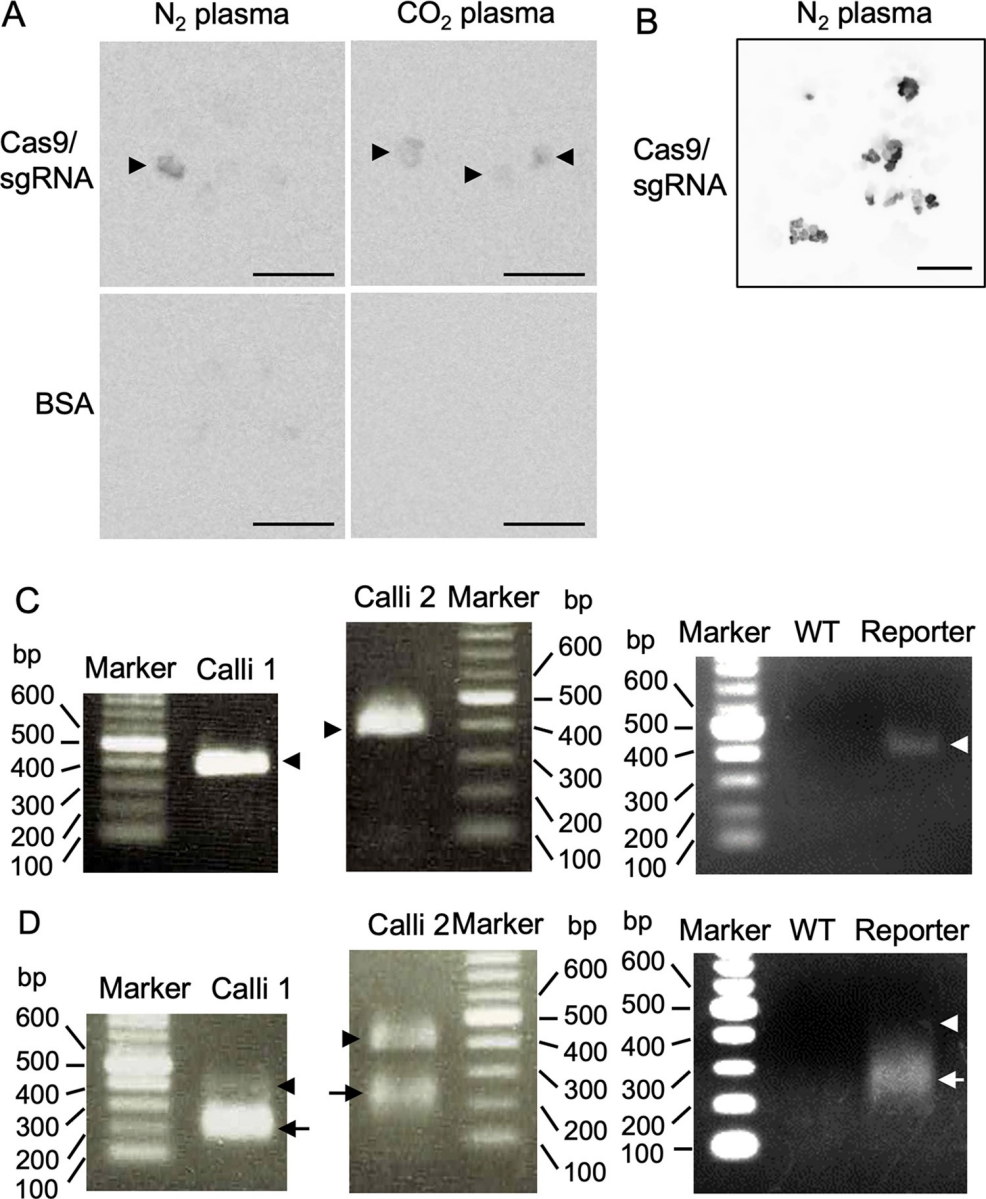
Detection of rice cells with edited genomes. (A) After calli introduced by Cas9/sgRNA or BSA were cultured on N6D medium for 5 days, they were treated with luciferin. Luminescence was detected in a part of calli introduced by Cas9/sgRNA but not BSA. Arrowheads indicate luminescent signals. Bars; 0.5 cm. (B) Luminescent calli were concentrated by culturing in a new medium and selection of luminescent calli every 2 weeks for 2 months. Bar; 1 cm. (C) Parts of CaMV 35S promoter and *LUC* ORF comprising the I-*Sce*I recognition sequence were amplified via PCR from genome DNA extracted from the independent luminescent calli (Calli 1 and Calli 2), untreated calli with (Reporter) and without (WT) the *L-(I-SceI)-UC* reporter gene. The amplified fragments were observed by agarose gel electrophoresis. Note that no detectable band was obtained in wild type (WT), indicating that the fragment was amplified from the reporter gene. Arrowheads indicate the PCR fragments. (D) The amplified samples were digested by I-*Sce*I. Genome editing by Cas9/sgRNA produces I-*Sce*1 resistant fragment. The digested samples were separated by agarose gel electrophoresis. I-*Sce*I digested bands are shown by arrows. Arrowheads indicate fragments that could not be cut by I-*Sce*I.

To evaluate genome editing in reporter rice, rice calli were irradiated with N_2_ or CO_2_ plasma and soaked in a solution containing Cas9/sgRNA for *L-(I-SceI)-UC* gene, as shown in Fig 2B. If genome editing occurred, calli with chemiluminescent ability may appear after cultivation. As shown in Fig 3A, calli with LUC luminescence appeared after N_2_ and CO_2_ plasma treatment, followed by soaking in Cas9/sgRNA solution. However, faint or no signal was detected in the calli soaked in the solution with the negative control BSA after N_2_ or CO_2_ plasma treatment. These results implied that the reporter gene was edited upon introduction of Cas9/sgRNA into the reporter rice using plasma treatment. To concentrate calli with luminescent signals, the calli introduced with Cas9/sgRNA by N_2_ plasma, which exhibited chemiluminescence, were picked and transferred to a new medium plate every two weeks. Repeated cultivation resulted in calli with strong luminescence signals (Fig 3B).

**Fig 4:**
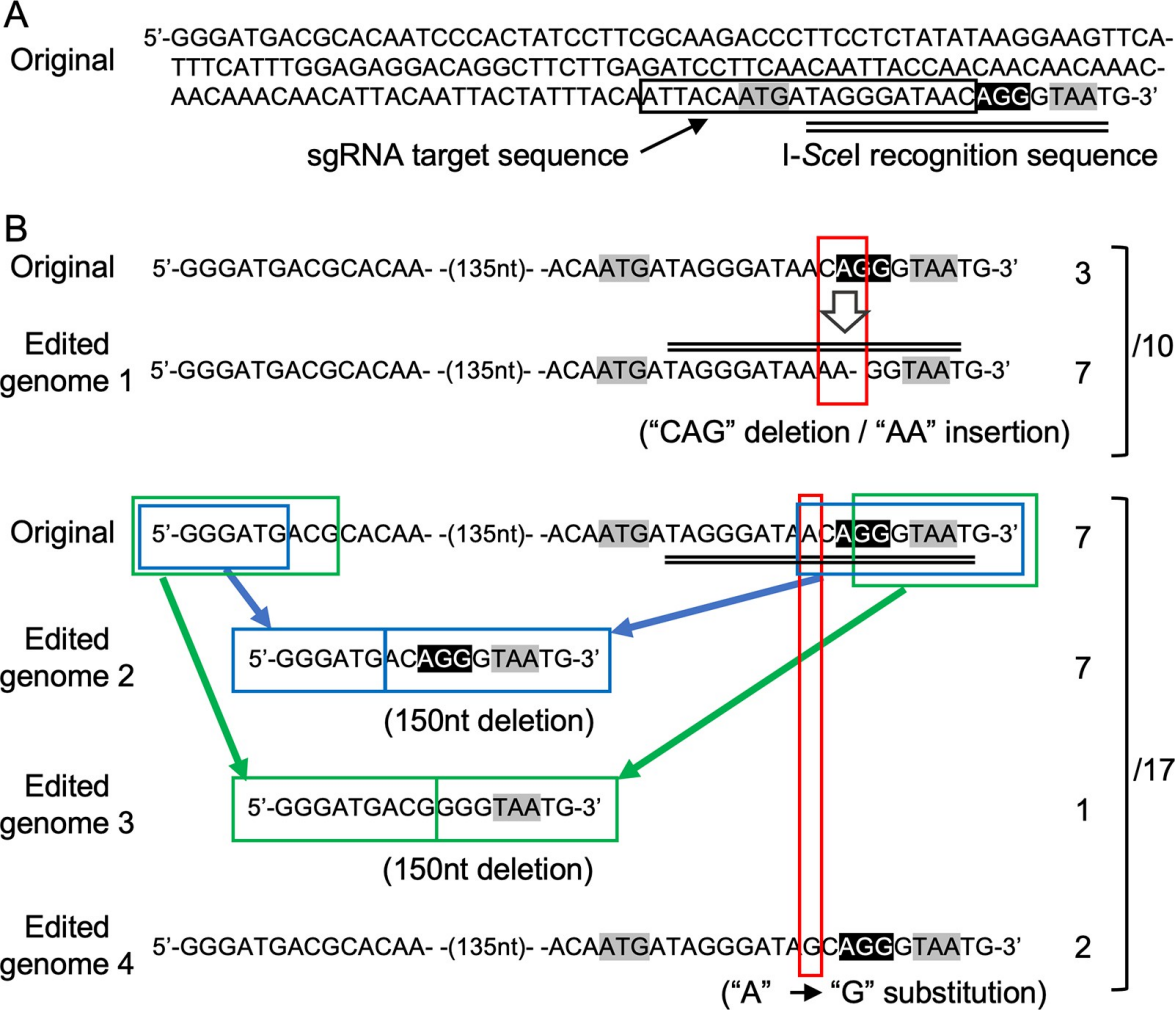
Genome-edited sequences derived from rice calli.

To confirm genome editing, a DNA fragment containing the target sequence of Cas9/sgRNA was amplified by PCR and assayed using the cleaved amplified polymorphic sequence, cloned, and sequenced. DNA fragments containing the presumed genome-edited region (parts of the CaMV 35S promoter and *LUC* ORF containing the I-*Sce*I recognition sequence; approximately 400 bp in length) were amplified from independently plasma-treated calli (Fig 3C left and middle). The resultant fragments were digested with I-*Sce*I to distinguish genome-edited fragments and fragments derived from the original sequence (Fig 3D). Since the I-*Sce*I recognition sequence was disrupted by DNA cleavage and subsequent repair errors, the appearance of the I-*Sce*I resistant band indicated the occurrence of genome editing at the target sequence. The genome-edited DNA fragment that could not be cut by I-*Sce*I most likely had a size similar to that of the amplified fragment; however, it may have undergone considerable insertion or deletion. As shown in Fig 3D, I-*Sce*I-resistant DNA fragments were obtained as bands of approximately 400 bp in the left and middle panels. Unlike them, a band of approximately 400 bp from the untreated reporter (Fig 3C right) was completely digested by I-*Sce*I in the right panel of Fig 3D, suggesting that the undigested fragments were produced by genome editing. To sequence the undigested bands, each digested sample was cloned into the cloning vector, as described in the Materials and Methods, and subsequent *E*. *coli* colonies were selected. Ten or 17 colonies were selected to prepare plasmid DNAs and for reading the sequences of DNA fragments in samples derived from calli 1 and 2, respectively, as shown in Fig 3D. The sequencing data are shown in Fig 4. A sequence with the deletion of 3 base pairs (CGA) and insertion of 2 base pairs (AA) was identified and designated as edited genome 1, which was observed in 7 plasmid DNA sequences out of the 10 plasmids isolated from calli 1 shown in Fig 3D. The remaining 3 plasmid DNA contained the original sequence. Three distinct DNA fragments with 2 types of 150 bp deletions and1 base pair substitution (A to G) were obtained from 7 (edited genome 2), 1 (edited genome 3), and 2 (edited genome 4) plasmid DNA out of 17 plasmids isolated from calli 2 (Fig 3D), implying the possibility of multiple genome-edited cells in the calli. The remaining 7 plasmid DNA contained the original sequence.

The DNA fragments in the I-*Sce*I digested samples (Fig 3D) were sequenced for comparison with the original sequence in the predicted genome-edited region. The original sequence is shown in (A). The sgRNA target sequence, start codons (ATG), PAM sequences (AGG), and stop codons (TAA), corresponding to those in Fig 2A, are indicated in squares. Double line indicates I-*Sce*I recognition sequence. (B) Four types of genome-edited sequences. Edited genome 1 was a sequence obtained from the I-*Sce*I digested sample of Calli 1. Edited genomes 2–4 were sequences obtained from the I-*Sce*I digested sample of Calli 2. Upon analysis of the ten sequences derived from Calli 1, seven genome-edited sequences (edited genome 1) and 3 original sequences were obtained (marked as “7/10” on the right). Upon sequence analysis of seventeen sequences derived from Calli 2, seven (edited genome 2), 1 (edited genome 3), or 2 (edited genome 4) genome-edited sequences and seven original sequences were obtained (marked as 7/17, 1/17, or 2/17). The presumed organizations of genome-edited sequences are indicated in parentheses. “nt” refers to nucleotide.

### Genome editing of tobacco leaf pieces carrying a reporter gene

**Fig 5:**
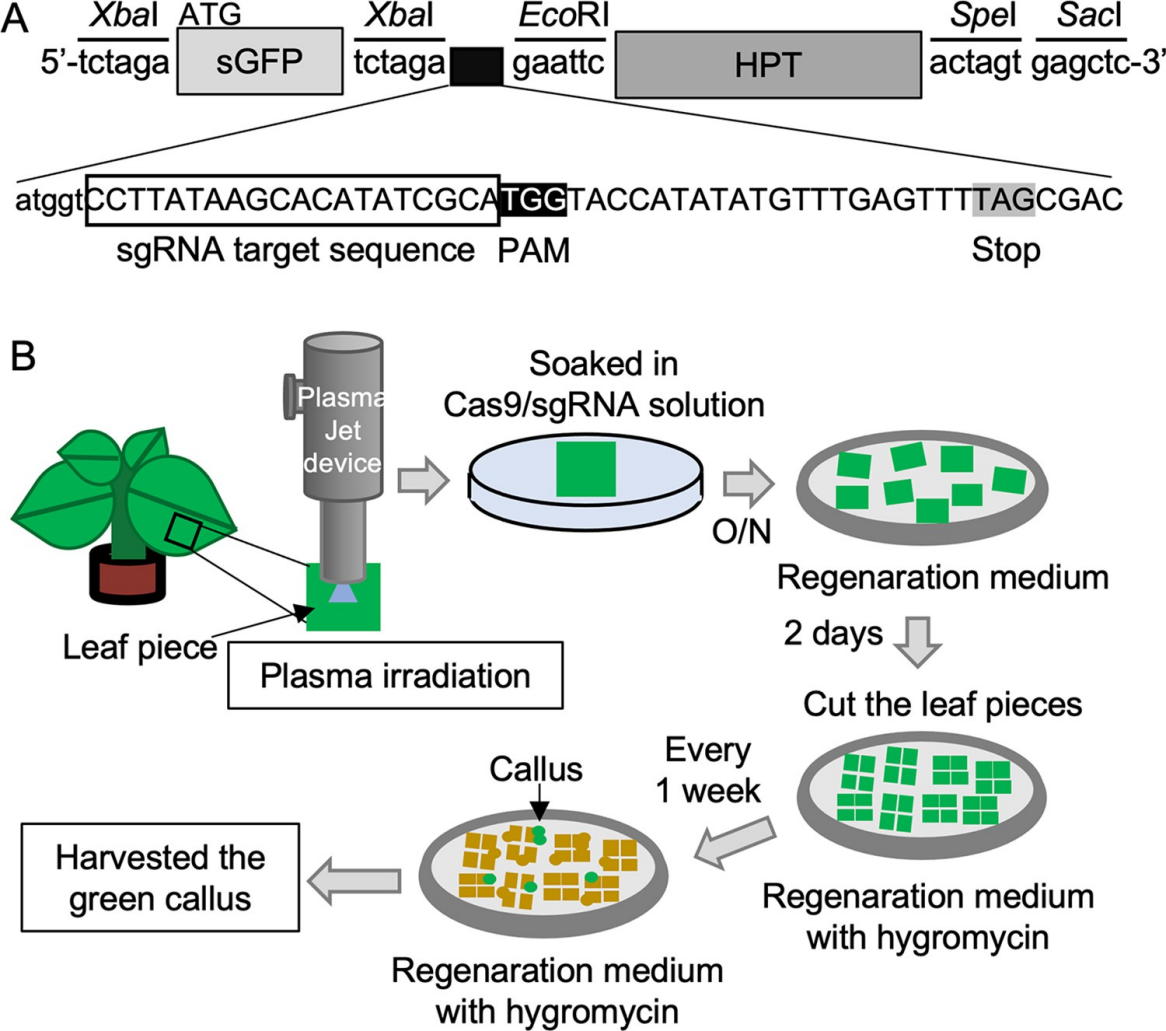
Development of tobacco reporter plant and procedure of plasma treatment. (A) Structure of *sGFP-waxy-HPT* reporter gene. *sGFP*, *waxy* gene (Capital letters), and *HPT* sequences were connected using restriction enzymes (*Xba*I, *EcoR*I, *Spe*I, and *Sac*I) and inserted into the pBI121 vector as described in Materials and Methods. A start codon of *sGFP* was used for translation. In the original construct, only sGFP-waxy is translated, and the *HPT* gene is not translated because the translation is stopped at stop codon (TAG) located upstream of the *HPT* gene in *waxy* gene sequence, producing no hygromycin resistance. sgRNA target sequence was designated as that comprising 20 nucleotides located immediately upstream of PAM sequence (TGG). Cas9/sgRNA digests at a site located approximately 3 to 4 base pairs upstream of the PAM sequence. *HPT* sequence can be changed in-frame when a frameshift occurs upon misrepair after digestion by Cas9/sgRNA, leading to the translational fusion of sGFP-waxy-HPT, which can exhibit hygromycin resistance. (B) Scheme of generating genome-edited tobacco plants by introducing Cas9/sgRNA into the cells using plasma. A leaf piece of sGFP-waxy-HPT reporter tobacco was irradiated by N_2_ plasma. The treated leaf piece was soaked in Cas9/sgRNA or BSA solution and kept overnight (O/N). The leaf pieces were transferred to regeneration medium plate and maintained for 2 days. The leaf piece was cut into 4 pieces and transferred on the regeneration medium plate containing hygromycin. The leaf pieces were transferred to new regeneration medium plate containing hygromycin every week. Calli appeared on regeneration medium plate upon cultivation of tobacco leaf pieces. Since green calli should be hygromycin resistant, a green callus was harvested for further analysis.

To assess whether genome editing via plasma treatment was successful in tobacco, a reporter tobacco plant carrying the reporter gene *sGFP-waxy-HPT* was developed as an evaluation system (Fig 5A). In the reporter gene, the hygromycin-resistant gene, *HPT*, was located downstream of the ORF of *sGFP*, which is a spacer to strongly suppress the expression of hygromycin-resistant gene without genome editing. The ORF of *HPT* was designed to avoid translation by inserting a *waxy* gene sequence with a stop codon between *sGFP* and *HPT*, so that the original plant could not exhibit hygromycin resistance. If Cas9/sgRNA digested at a site located upstream of the PAM sequence and insertion or deletion occurred, the in-frame shift of the *waxy* gene sequence would produce an sGFP-fused HPT protein. This would result in hygromycin resistance upon the translation of the *HPT* gene located downstream of *sGFP*. Therefore, calli with genome-edited sequences should appear upon cultivating leaf pieces with genome-edited cells in hygromycin-containing regeneration medium (Fig 5B).

**Fig 6:**
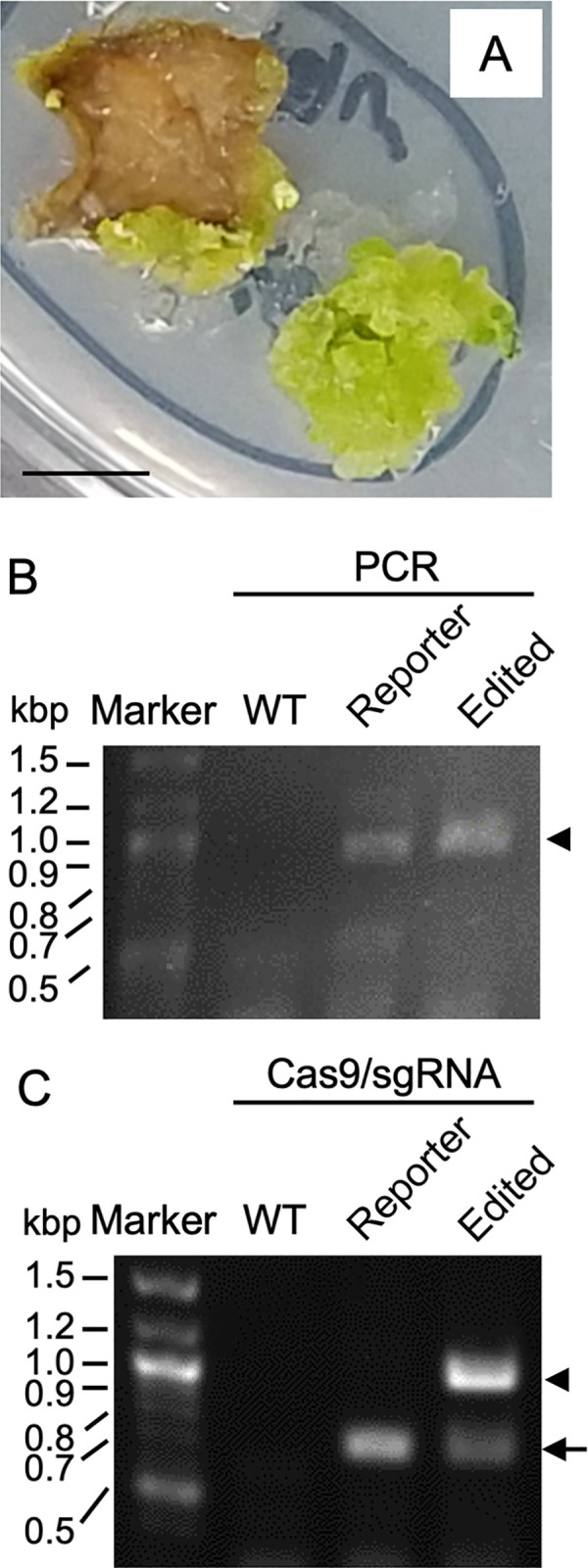
Detection of genome-edited tobacco cells. (A) A green callus appeared in the regeneration medium in the presence of hygromycin. Bar; 1 cm. (B) Parts of *sGFP* and *HPT* containing the *waxy* sequence were amplified by PCR from genomic DNA extracted from the callus shown in (A) (Edited), untreated tobacco leaf with (Reporter) and without (WT) the *sGFP-wTALEN-HPT* reporter gene. The amplified fragment was shown by agarose gel electrophoresis. Note that no detectable band was obtained in WT, indicating that the fragment was amplified from the reporter gene. An arrowhead indicates the PCR fragments. (C) Genome editing by Cas9/sgRNA produced resistant fragments for Cas9/sgRNA digestion. The PCR product was digested with Cas9/sgRNA and separated by agarose gel electrophoresis. An arrowhead indicates a fragment that could not be cut by Cas9/sgRNA. Cas9/sgRNA digested bands are shown by an arrow.

As shown in rice experiments and previous reports [13], both N_2_ and CO_2_ plasmas are similarly effective to introduce macromolecules. Thus, to generate genome-edited reporter tobacco plants, the leaf piece was irradiated with N_2_ plasma and soaked in a solution containing Cas9/sgRNA, as shown in Fig 5B. As shown in Fig 6A, hygromycin-resistant calli appeared in leaf pieces soaked in Cas9/sgRNA solution after N_2_ plasma treatment.

**Fig 7:**
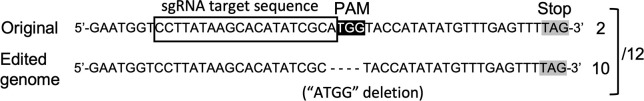
Genome-edited sequences derived from a tobacco callus.

To confirm whether genome editing occurred, a DNA fragment containing the presumed genome-edited region (parts of *sGFP* and *HPT* containing the *waxy* gene sequence; approximately 1.05 kbp in length) was amplified from the green callus (Fig 6B), and the produced fragment was digested with Cas9/sgRNA (Fig 6C). Once genome editing occurred, the edited DNA could no longer be digested by Cas9/sgRNA. Thus, the appearance of DNA fragments that could not be cut by Cas9/sgRNA suggested successful genome editing. As shown in Fig 6C, Cas9/sgRNA-resistant DNA fragment was observed as a band of approximately 1.05 kbp in hygromycin resistant callus, but not in the untreated reporter tobacco. For sequencing, the digested samples were cloned into vectors using the same method as that used for L-(I-SceI)-UC rice. Twelve colonies were selected to prepare the plasmid DNAs and read the sequences of the DNA fragments containing the presumed genome-edited region. The sequencing results are shown in Fig 7. A sequence with a deletion of 4 base pairs was observed in 10 out of 12 plasmids. The remaining 2 plasmid DNAs demonstrated the original sequence.

The genome-edited sequence was obtained from the sample shown on Fig 6C. The sgRNA target sequence, PAM sequences (TGG), and stop codons (TAG) corresponding to Fig 5A are shown in squares. Dashes correspond to the number of nucleotides deleted via genome editing. Upon examination of the 12 sequences, ten genome-edited sequences and 2 original sequences were observed (marked as “10/12” on the right). The presumed organization of the genome-edited sequence is indicated in parentheses.

To examine whether the genome-edited trait is inherited, six regenerated plants were obtained from the hygromycin-resistant callus (Fig 6A) and their seeds were harvested. As shown in S1 Fig and Table 1, the ratio of hygromycin-resistant and sensitive seedlings was approximately 2:1 to 7:1 (average 3.8:1) compared to no resistant ones in untreated reporter plants. Same sequence (ATGG deletion) to Edited genome in Fig 7 was obtained in the genome prepared from a hygromycin-resistant seedling from seed number 18801 (Table 1 and S1 Fig). These results indicate that the trait produced by genome editing should be inherited by next generation.

**Table 1:** Hygromycin resistance was examined in tobacco seedlings from seeds of untreated reporter or regenerated genome-edited plants. Number of seedlings in S1 Fig was counted.

Plant type	Seed name	Number of seedlings		Ratio	
		Resistant	Sensitive	Resistant	Sensitive
Reporter	14830	0	50		
Genome-edited	18801	36	14	2.6	1
	18802	33	17	1.9	1
	18803	38	12	3.2	1
	18804	38	12	3.2	1
	18805	41	9	4.6	1
	18806	44	6	7.3	1

## Discussion

In this study, we succeeded in genome editing of reporter genes in rice and tobacco plants by directly introducing Cas9/sgRNA into the plasma-treated cells. Plants, unlike mammalian cells, have specific structures, such as wax and cuticular layers, rendering direct introduction of Cas9/sgRNA difficult into the cells of intact tissues. Therefore, in many cases, genetic transformation has been used for genome editing in plants. To obtain genome-edited plants, transgenes should be removed after genome editing. In the case of seed-propagated plants, null segregants can be obtained by Mendelian segregation; however, the development may take a long time in some cases. Since plants reproducing via vegetative-propagation, such as potato and strawberry, are heterogeneous, their next generation exhibits different characteristics. Therefore, null segregants of such plants with the same characteristics cannot be obtained. Since proteins and RNA are degraded in the introduced cells, directly introduced genome editing enzymes do not need to be removed from the cells. Thus, genome editing by direct introduction of Cas9/sgRNA in plant cells is especially useful in plants that reproduce via vegetative propagation. In such plants, removing transgenes genetically is difficult, adding to the advantage of genome editing in them.

It is known that methods using CPP and in planta particle bombardment have worked for CRISPR/Cas9-mediated genome editing in plants [10, 12]. However, there are limitations on the tissues that can be used, such as shoot meristems, and/or need for pretreatment for introduction of genome editing enzymes. A bacterium carrying a type III secretion system is useful for protein- based genome editing enzymes, such as TALEN [11]. However, it can transfer only proteins into the cells and have demonstrated certain limitations with respect to its application in host plants. Previously, we showed that sGFP fusion protein could be introduced into the cells of intact *Arabidopsis* leaves and rice roots, in addition to rice calli and tobacco leaves by plasma method without any pretreatment [13]. Together with the introduction of Cas9/sgRNA in this study, our temperature-controlled atmospheric pressure plasma demonstrated possible application in various plant species and tissues. The molecular weight of used Cas9 or sgRNA was approximately 150,000 or 20,000, respectively. In addition, pUGW2 plasmid carrying *sGFP* gene, which molecular weight is approximately 4,000,000, could be introduced into plant cells by plasma treatment [22]. Thus, we believe that various macromolecules such as protein, RNA and DNA with a molecular weight of at least 4,000,000 or less can be introduced into plant cells using our plasma-treatment method. Thus, it is also expected that our method can be used for genome editing using other genome editing enzymes, such as TALEN, in plants. Considering the characteristics of this plasma-treatment method, it can be useful for genome editing in various tissues and plant species using any genome editing enzymes. Therefore, this method is expected to accelerate genome editing in plants, with wide applications in plant breeding in the future.

In this study, we showed that treatment with N_2_ or CO_2_ plasma aided in genome editing upon introduction of Cas9/sgRNA into plant cells. Previously, we judged that N_2_ or CO_2_ plasma was more effective and easier to use than other types of plasma for introduction of sGFP fusion protein into plant cells [13]. Hence, we used N_2_ or CO_2_ plasma in this study. It was reported that Ar plasma worked to introduce Cas9/sgRNA in mammalian cells [14]. We also showed that an sGFP fusion protein was introduced into plant cells upon treatment with Ar plasma, although the results observed using Ar plasma were not better than those observed using N_2_ or CO_2_ plasma [13]. The mechanism underlying introduction of proteins by plasma into cells via endocytosis is almost similar in both plants and mammalian cells; however, previous studies have used Ar or air plasma for the treatment of mammalian cells [14, 23, 24]. These results imply that plasma generated by other gases, such as Ar, may work to introduce Cas9/sgRNA into plant cells, although plasma generated from different gas sources produce qualitatively and quantitatively distinct reactive species that can influence protein uptake [25, 26]. Whether treatment with plasma generated from other gases may work for CRISPR/Cas9-mediated genome editing in plants will be elucidated in future.

## Conclusions

In this study, genome editing was achieved by introducing Cas9/sgRNA directly into plant cells using temperature-controlled atmospheric pressure plasma.

Genome editing was successfully performed using both calli and leaf pieces as plant materials for introduction of Cas9/sgRNA using the same plasma treatment method. Both N_2_ and/or CO_2_ plasma was effective for introduction of Cas9/sgRNA for genome editing in rice calli and tobacco leaf pieces at a flow rate of 5 L/min and a treatment time of 5 sec. The temperature of plasma was approximately 20 to 30°C, which was similar to that during the introduction of sGFP fusion protein [13].

To evaluate genome editing, two reporter genes, *L-(I-SceI)-UC* and *sGFP-waxy-HPT*, were used in rice calli and tobacco plants, respectively. Four types of genome-edited sequences were obtained from the presumed genome-edited region of *L-(SceI)-UC* in rice calli. In tobacco, a genome-edited sequence was obtained from the presumed genome-edited region of s*GFP-waxy-HPT* in tobacco callus produced from a plasma-treated leaf piece. These results certified that Cas9/sgRNA was introduced into rice and tobacco cells by plasma treatment, and successful genome editing was performed by introducing Cas9/sgRNA into rice and tobacco.

## Supporting information

S1 Fig Hygromycin resistance was examined in the regenerated plants from a genome-edited tobacco callus. Seeds from untreated reporter (Reporter) or 6 regenerated plants obtained from the callus in Fig 6A (Genome-edited) were sowed on hygromycin containing medium plates and cultured for 2 weeks. Bars; 1 cm.
(TIF)

S1 Table Primers and oligos used in this work. (PDF)
