# Supplementary figures and images for: Genome editing by introduction of Cas9/sgRNA into plant cells using temperature-controlled atmospheric pressure plasma
Source: PLoS One. 2023 Feb 16;18(2):e0281767. doi: 10.1371/journal.pone.0281767 (PMC9934431; doi:10.1371/journal.pone.0281767)

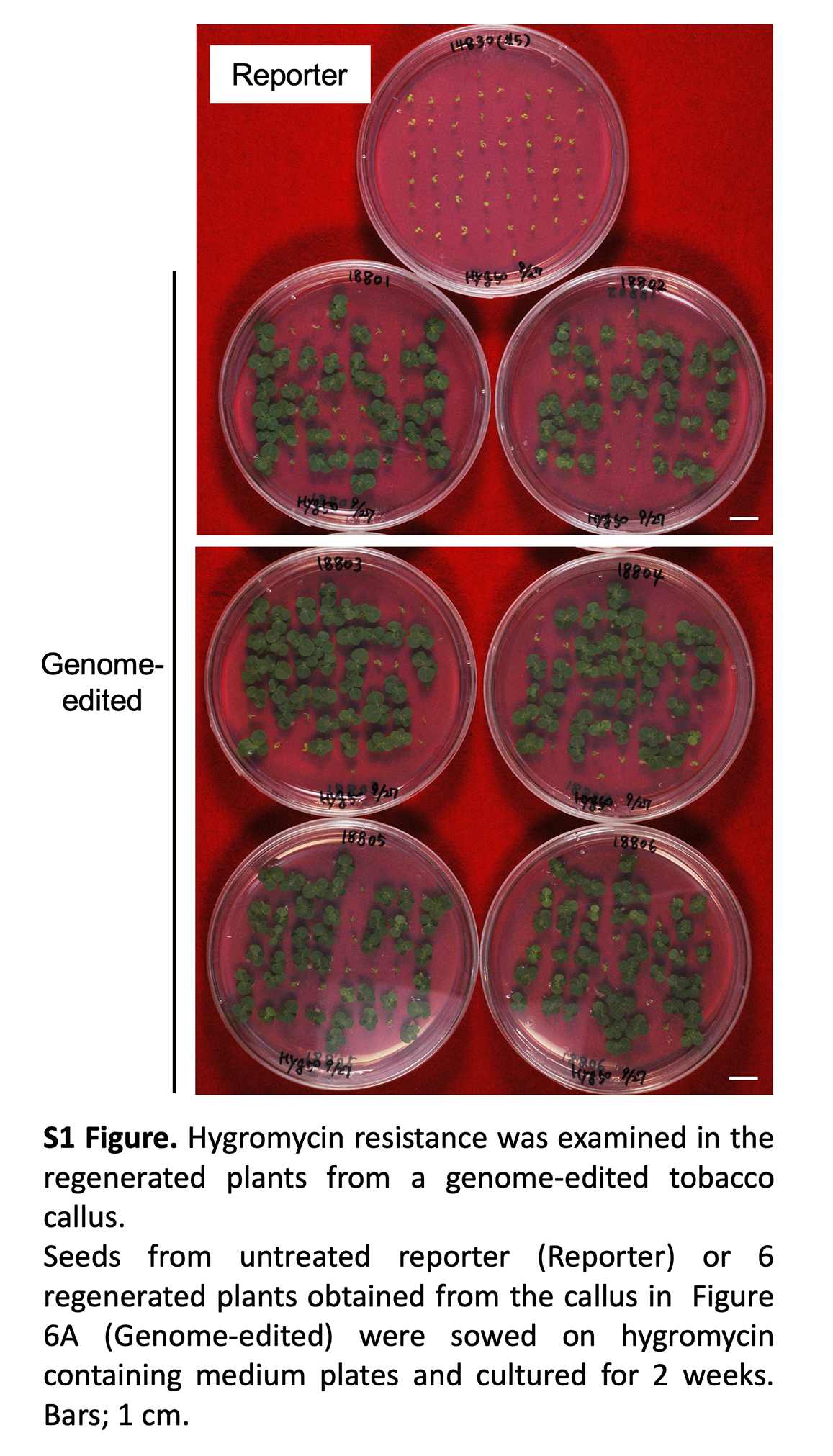

Supplement: S1 Fig — Seeds from untreated reporter (Reporter) or 6 regenerated plants obtained from the callus in Fig 6A (Genome-edited) were sowed on hygromycin containing medium plates and cultured for 2 weeks. Bars; 1 cm. (TIF) [file pone.0281767.s001.tif]
