# Supplementary material for: Genome editing by introduction of Cas9/sgRNA into plant cells using temperature-controlled atmospheric pressure plasma
Source: PLoS One. 2023 Feb 16;18(2):e0281767. doi: 10.1371/journal.pone.0281767 (PMC9934431; doi:10.1371/journal.pone.0281767)
Supplement: S1 Table — (PDF) [file pone.0281767.s002.pdf]

S1 Table. Primers and oligos used in this work

Name	5' - 3'
XbaI-waxy-EcoRI-F	CTAGAATGGTCCTTATAAGCACATATCGCATGGTACCATATATGTTTGAGTTTTAGCGACG
EcoRI-waxy-XbaI-R	AATTCGTCGCTAAAACTCAAACATATATGGTACCATGCGATATGTGCTTATAAGGACCATT
cHPT-F	CATGAAAAAGCCTGAACTCACCGC
SpeI-HPT-R	ATACTAGTTTATGGAGAAACTCGAGCTTGATAAG
XbaI-sGFP-F	AGTCTAGAATGGTGAGCAAGGGCGAGG
XbaI-sGFP-R	AGTCTAGACTTGTACAGCTCGTCCATGC
Guide-it scaffold template	GCACCGACTCGGTGCCACTTTTTCAAGTTGATAACGGACTAGCCTTATTTTAACTTGCTA
GFP-wx Guide it	TGCGGCCTCTAATACGACTCACTATAGGGCCTTATAAGCACATATCGCAGTTTTAGAGCT
I-Sce1-LUC Guide it	TGCGGCCTCTAATACGACTCACTATAGGGATTACAATGATAGGGATAACGTTTTAGAGCT
P35s-90-5	ATCTCCACTGACGTAAGGGATGACG
ELUC-217R	TCATCTTGTAGCCACAGTTCT
sGFP-441F	CAGCCACAACGTCTATATC
HPT3	TCGCCTCGCTCCAGTCAATG
